# Effects of soil pH and texture on soil carbon and nitrogen in soil profiles under different land uses in Mun River Basin, Northeast Thailand

**DOI:** 10.7717/peerj.7880

**Published:** 2019-10-15

**Authors:** Wenxiang Zhou, Guilin Han, Man Liu, Xiaoqiang Li

**Affiliations:** Institute of Earth Sciences, China University of Geosciences (Beijing), Beijing, China

**Keywords:** Soil carbon and nitrogen, Soil texture, Soil pH, Land use, Mun River Basin, Northeast Thailand

## Abstract

Soil carbon and nitrogen are essential factors for agricultural production and climate changes. A total of 106 soil samples from three agricultural lands (including two rice fields and one sugarcane field) and four non-agricultural lands (including two forest lands, one wasteland and one built-up land) in the Mun River Basin were collected to determine soil carbon, nitrogen, soil pH, soil particle sizes and explore the influence of pH and soil texture on soil C and N. The results show that total organic carbon (TOC) and nitrogen (TON) contents in topsoil (TOC: 2.78 ~ 18.83 g kg^−1^; TON: 0.48 ~ 2.05 g kg^−1^) are much higher than those in deep soil (TOC: 0.35 ~ 6.08 g kg^−1^; TON: <0.99 g kg^−1^). In topsoil, their contents of forest lands and croplands (TOC: average 15.37 g kg^−1^; TON: average 1.29 g kg^−1^) are higher than those of other land uses (TOC: average 5.28 g kg^−1^; TON: average 0.38 g kg^−1^). The pH values range from 4.2 to 6.1 in topsoil, and with increase in soil depth, they tend to increase and then decrease. Soil carbon, nitrogen and the C/N (TC/TN ratio) are negatively correlated with soil pH, demonstrating that relatively low pH benefits the accumulation of organic matter. Most soil samples are considered as sandy loam and silt loam from the percentages of clay, silt and sand. For soil profiles below 50 cm, the TOC and TON average contents of soil samples which contain more clay and silt are higher than those of other soil samples.

## Introduction

Soil holds more than three times as much carbon as the atmosphere and terrestrial vegetation, thus playing an important role in global climate change and agricultural production (Lal, 2004; Li et al., 2017). It is widely acknowledged that soil mitigates climate change by assimilating atmospheric carbon dioxide and converting it into soil carbon by means of total organic carbon (TOC) sequestration (Minasny et al., 2017). Total organic carbon is important for agricultural production because organic matter helps to improve soil structure and capacity of exchanging cation and holding water, thus exerting positive impacts on soil fertility (West & Post, 2002; De Blécourt et al., 2019; Liu et al., 2019). Numerous studies reveal that the concentration of TOC is affected by many factors; for example, the change of land use type is an important driving force for TOC dynamics by altering organic carbon input and decomposition rates (Zhang, Wang & Wang, 2014; Poeplau et al., 2011). Soil pH also influences TOC contents significantly, because it regulates soil nutrient bioavailability, organic matter turnover and an array of soil processes (Kemmitt et al., 2006; Robson, Snowball & Robson, 1989). Soil texture, which is used to describe the size distribution of mineral particles, is reported as another important factor influencing the accumulation soil organic matter (Dexter, 2004). Generally, clay and silt particles protect soil organic matter by stabilizing them against microbial mineralization (Six et al., 2002). Besides soil carbon, soil nitrogen is also important in sustaining soil quality, crop production and environmental protection (Doran & Parkin, 1994). Therefore, it’s important to learn about soil carbon and nitrogen as well as the relationship between them and other soil properties.

According to statistical data (Prabnakorn et al., 2018), Thailand has almost the lowest rice yield among Southeast Asian countries although being one of the largest rice-producing countries. Moreover, the average rice yield of the northeastern region (where the Mun River Basin is located) ranks at the bottom among other regions in Thailand. On the other hand, conversion between natural vegetation and cropland may influence many ecological processes and cause substantial changes in soil carbon, nitrogen and many other soil properties (Yu et al., 2014). Therefore, it’s vital to learn about the distribution characteristics of soil C and N and explore the influence of other soil physicochemical properties (including C/N ratio, soil pH and soil texture) on soil C and N under different land uses. Several studies have reported the distribution characteristics of soil organic matter, soil pH and soil electric conductivity the Mun River Basin (Zhao et al., 2018b; Hossain et al., 2017). However, most studies only focused on the topsoil without exploring the profile distribution characteristics of these properties.

Therefore, the aims of this study are as follows: (1) to determine the vertical distribution characteristics of soil C, N, C/N, pH and soil particle sizes; (2) to recognize the difference between these properties based on different land uses; (3) to explore the influence of pH and soil texture on soil C and N.

## Sampling and analytical procedures

### Study area

**Figure 1:**
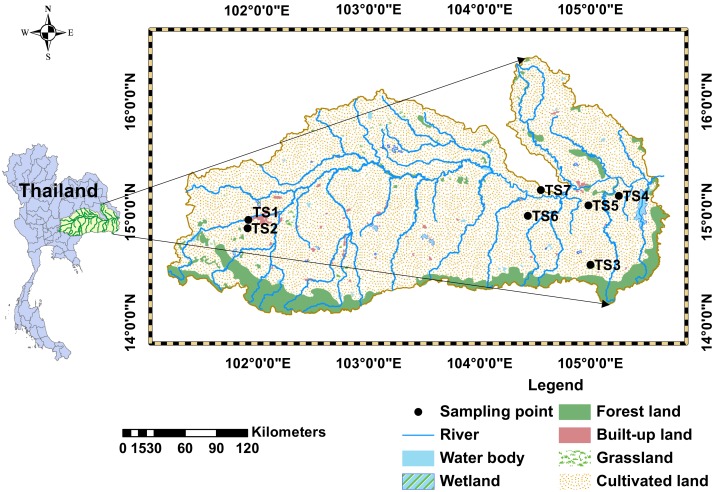
Map of soil sampling locations and land use types in the Mun River Basin.

The study area is within the Mun River Basin, which is located in northeastern Thailand (14°00′−16°00′N, 101°30′−105°30′E) and occupies an area of 82,000 km^2^ (Fig. 1). There are large areas of cultivated lands and forest lands. Other types of lands, such as wet lands, built-up lands, grasslands, etc., are sporadically distributed on the cultivated lands (Fig. 1). The Mun River Basin has a tropical savanna climate which consists of a dry season and a rainy season all year round, with the average annual rainfall of 1,300–1,500 mm (Wijnhoud, Konboon & Lefroy, 2003). Soil organic matter contents in the northeast Thailand are relatively low, therefore, chemical fertilizers, as well as organic fertilizers, are used to increase soil fertility (Khunthasuvon et al., 1998).

The Mun River Basin is mainly occupied by clastic sedimentary rocks of Mesozoic continental red beds known as Khorat Group, which consists of siltstone, sandstone, conglomeritic sandstone and shale (Nimnate et al., 2017). For example, the Khorat Plateau is occupied by Triassic sandstones with scattered lava flows (Carling, 2009). Most areas of the Mun River Basin are covered by unconsolidated Quaternary alluvial sediment (Dheeradilok, 1993).

### Soil sampling and analysis

Samples (total 106) were collected from seven soil profiles, including three agricultural lands (TS1, TS2, TS4) and four non-agricultural lands (forest land: TS3, TS7; wasteland: TS5; built-up land: TS6). It should be noted that sampling point TS4 is located in a rice field which was abandoned for 2 years. Weeds and trees of the wasteland (where TS5 is located) were burned in preparation for farming. These soil profiles were chosen to explore soil C, N, pH and soil texture in agricultural lands to compare with those in non-agricultural lands (Fig. 1). Soil samples for each profile were collected at 10 cm depth intervals. The detailed information of each soil profile is shown in Table 1.

**Table 1:** General description of seven soil profiles.

Sampling point	Latitude	Longitude	Land use type	Description
TS1	14°56′32.59″	101°58′34.10″	Rice fields	0–220 cm: Brown soil; no change in soil horizon observed
TS2	14°52′06.89″	101°57′58.69″	Sugar cane field	0–30 cm: Brown clay; compact and hard soil; massive structure
TS3	14°33′5.03″	104°56′14.76″	Oak field	0–18 cm: Humus layer
				18–50 cm: Reddish-brown subsandy soil
TS4	15°09′0.77″	105°10′56.44″	Rice fields	0–12 cm: Gray-black root layer; a few plant roots and wormholes
				12–47 cm: Fine silt
				47–100 cm: Light yellow fine silt; numerous brown and yellow spots of iron rust
				>100 cm: Iron manganese nodules layer; red black iron nodules
TS5	15°03′55.86″	104°55′7.50″	Wasteland	0–20 cm: Gray-black fine silt; planting layer
				20–40 cm: Grey fine silt; existing a few roots
				40–112 cm: Existing a small number of iron rusty spot and abundant iron and manganese nodules
				112–180 cm: Red weathering crust
				>180 cm: Bed Rock
TS6	14°58′33.11″	104°23′44.07″	Built-up land	0–20 cm: Fine silt; surface layer
				20–105 cm: Fine silt
				105–160 cm: Lime green sludge
				160–205 cm: Iron manganese tuberculosis layer
				205–405 cm: Lime green clay, lacustrine strata
				>405 cm: red siltstone
TS7	15°11′59.35″	104°30′28.23″	Forest	0–50 cm: Fine silt; lots of plant roots

After the removal of the plant residue, roots and stones, soil samples were ball milled to pass a 200-mesh sieve to make them homogenized with a grinding machine Retsch MM400 (Retsch GmbH, Haan, Germany). Total carbon (TC) and total nitrogen (TN) were measured with an elemental analyzer (Vario TOC cube; Elementar, Langenselbold, Germany) whose precision is C ± 0.1% and N ± 0.02%. The mixture of 1M KCl and 0.5M HCl was used to eliminate inorganic carbon and inorganic nitrogen (Kachurina et al., 2000; Midwood & Boutton, 1998). After being soaked with the mixture for 24 h, soil samples were washed with pure water repeatedly until they became neutral. Measured by the elemental analyzer again, total organic carbon (TOC) and total soil organic nitrogen (TON) were determined. Total inorganic carbon (TIC) was obtained by subtracting TOC from TC and total inorganic nitrogen (TIN) was obtained by subtracting TON from TN. After soil solutions (soil:water 1:2.5) were shaken and left to stand for 30 min, the pH was measured using a pH-meter (Navas et al., 2012), whose precision is ±0.05. Under heating conditions, 30% hydrogen peroxide was used to wipe out organic matter in soil and 10% HCl was used to wipe out soil carbonate. Finally, after dispersing these soil samples using ultrasounds physically, soil particle sizes were measured using a laser particle analyzer (Mastersizer 2000) (Ryżak & Bieganowski, 2011).

### Statistical analysis

Correlation analysis was conducted using SPSS 20 (Statistical Package for Social Science) and all figures are produced with OriginPro 9.0 software and CorelDRAW Graphics Suite 2018.

## Results

### Soil C, N, C/N and pH in surface layer

As shown in Table 2, soil pH is lower than seven at all sampling points, ranging from 4.2 to 6.1. Generally, pH values are little influenced by land use and they are relatively lower at the sampling points which are close to the central region of the Mun River Basin. In the surface layer, the C/N ratio ranges from 7.96 to 13.42. The lowest C/N ratio occurs at the sampling point TS6 which received little organic matter. Soil TC contents of forest lands (TS3: 19.98 g kg^−1^, TS7: 13.56 g kg^−1^) are similar with those of croplands (TS1: 18.11 g kg^−1^, TS2: 17.95 g kg^−1^). The TC contents are relatively lower at sampling points TS4, TS5 and TS6, in the range of 3.26–8.68 g kg^−1^. Soil TN, TOC, TON contents have similar distribution characteristics. One exception is TS7 has relatively lower TON content, just 0.56 g kg^−1^. Total inorganic carbon contents at TS4 and TS6 are much lower compared with those at other sampling points, while TIN contents at TS2 and TS4 are much lower compared with those at other sampling points.

**Table 2:** Soil C, N, C/N and pH in surface layer of seven soil profiles.

Land use type	pH	TC (g kg^−1^)	TN (g kg^−1^)	TOC (g kg^−1^)	TON (g kg^−1^)	TIC (g kg^−1^)	TIN (g kg^−1^)	C/N^1^
Rice fields	5.6	18.11	1.74	15.45	1.20	2.66	0.54	10.41
Sugar cane field	6.1	17.95	1.41	15.29	1.35	2.66	0.06	12.73
Oak field	5.6	19.98	2.29	18.83	2.05	1.15	0.24	8.73
Rice fields	5.7	5.85	0.65	5.62	0.59	0.23	0.06	9.00
Wasteland	5.5	8.68	0.81	7.45	0.48	1.23	0.33	10.72
Built-up land	4.3	3.26	0.41	2.78	0.08	0.48	0.33	7.96
Forest	4.2	13.56	1.01	11.92	0.56	1.64	0.45	13.42

**Note:**

1 C/N, TC to TN ratio.

### Vertical distribution of soil C, N, C/N and pH

**Figure 2:**
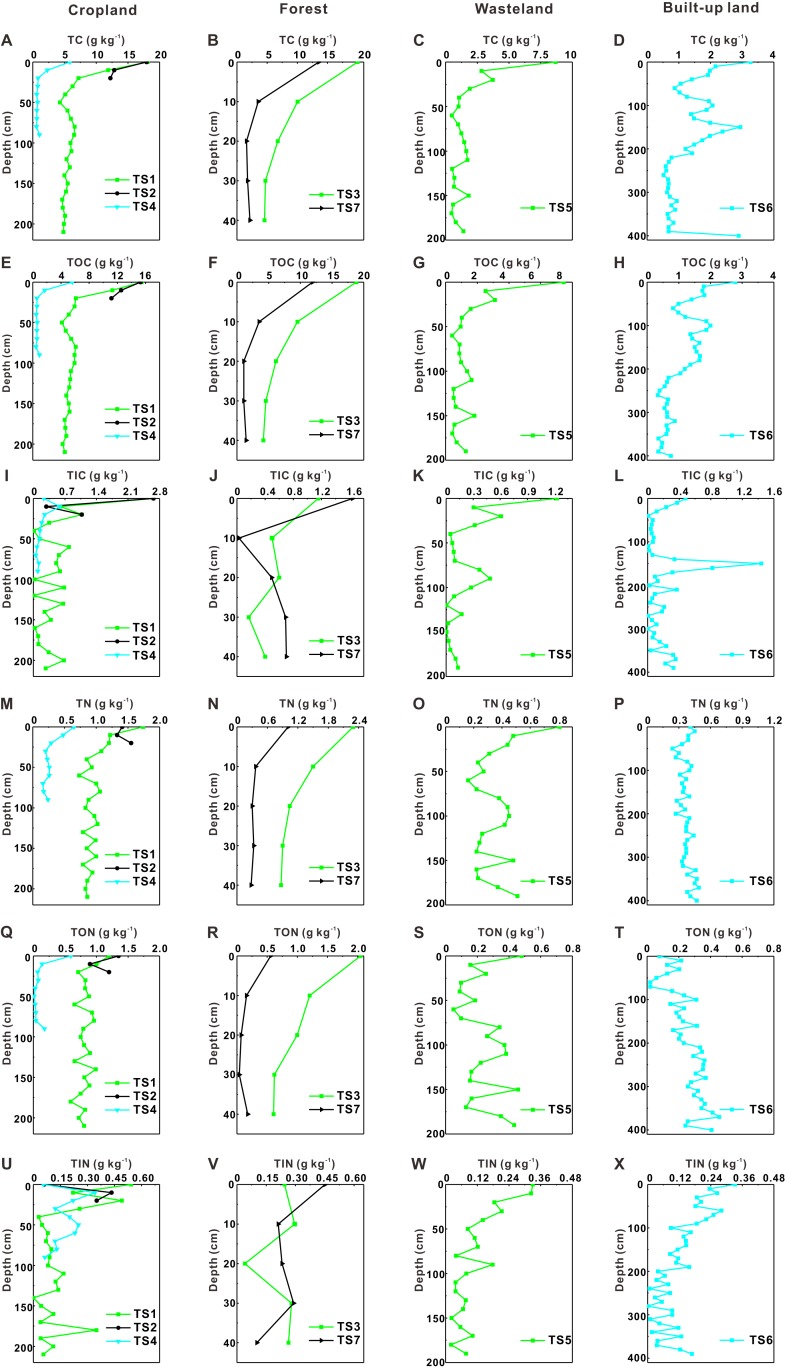
Profile distribution of soil carbon and nitrogen. (A–D) Profile distribution of total carbon; (E–H) profile distribution of total organic carbon; (I–L) profile distribution of total inorganic carbon; (M–P) profile distribution of total nitrogen; (Q–T) profile distribution of total organic nitrogen; (U–X) profile distribution of total inorganic nitrogen.

**Figure 3:**
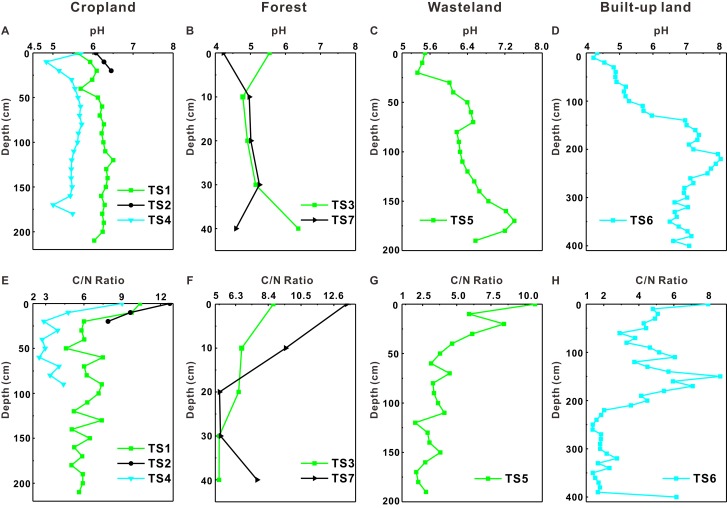
Profile distribution of soil pH and C/N. (A–D) Profile distribution of soil pH; (E–H) profile distribution of soil C/N.

Figure 2 presents profile distribution characteristics of soil carbon and nitrogen (including TC, TOC, TIC, TN, TON, TIN) in different soil profiles. The TC, TOC, TN, TON contents in soil profiles TS1, TS2 and TS3 are higher than those in other soil profiles (Fig. 2). Generally, the four variables (TC, TOC, TN, TON) of soil below 20 cm in most profiles show relatively uniform distribution but a sharp decrease occurs at around 20 cm depth. As for TIC and TIN, their contents for all soil profiles are similar with each other at all depths. Compared with TOC and TON contents, TIC and TIN contents change dramatically along with soil depth. As shown in Fig. 3, pH values tend to increase with depth generally. But they show positive peaks at the middle sections of most soil profiles (except for TS2), which is particularly obvious in TS4 and TS6. On the other hand, C/N values (TC to TN ratio) tend to decrease with depth, which is contrary to the distribution tendency of pH values.

### Soil texture

**Figure 4:**
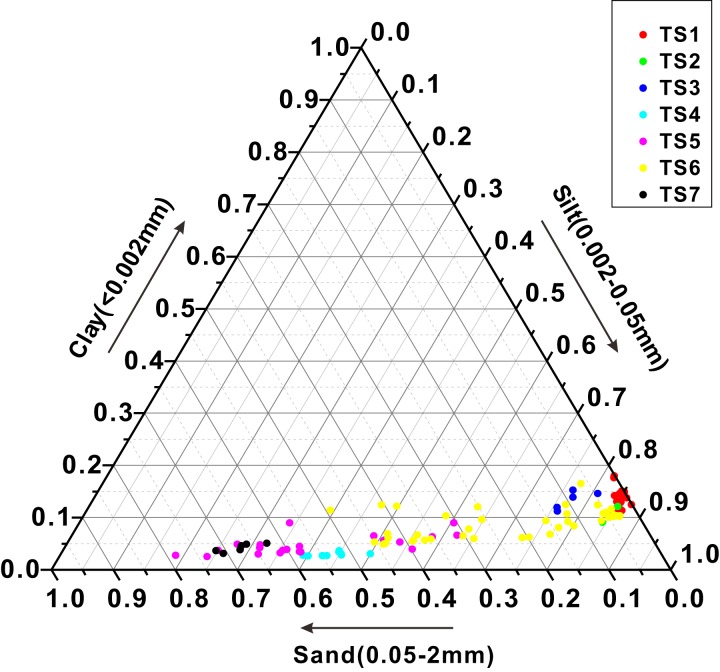
Soil textural triangle of all samples.

After the soil particle sizes are obtained, all soil particles are classified into three groups (clay, silt, sand) according to the soil classification system used in America (Staff Soil Survey, 2010). Percentages of clay, silt and sand of each soil sample are shown in Fig. 4. It shows that the percentages of clay are less than 25% for all samples in a narrow range, but the percentages of silt and sand vary largely among different samples. Soil samples from TS1, TS2 and TS3 have higher clay percentages than others. From the perspective of sampling points, clay, silt and sand contents of soil samples from sampling points TS1, TS2, TS3, TS4 and TS7 are relatively stable. But these contents of samples from sampling points TS5 and TS6 are in a relatively large range, indicating that soil texture of these soil samples is different from each other. Specifically, soil samples from TS1, TS2 and TS3 are silt loam and soil samples from TS4 and TS7 are sand and loamy sand, respectively. Soil samples from TS5 and TS6 include all these soil textures, which is consistent with our field observation. Soil profiles TS5 and TS6 have many distinctive soil layers which are different from each other in texture. The detailed description of these layers is shown in Table 1.

### Correlation between pH and soil C, N, C/N ratio

The correlation coefficients between pH and other soil properties at different sampling points are shown in Table 3. Soil TOC, TON, TIC, TIN and C/N ratio were chosen as representatives of various properties because they are essential factors in agricultural production (TC and TN were excluded because they were similarly distributed with TOC and TON). Most of these properties at different sampling points correlate well with soil pH, except for TS3 (Table 3). Relatively, the correlation between TON and pH is not as strong as other properties. In general, except for the properties which are not correlated with pH significantly, most properties are negatively correlated with pH. One exception is that TON at sampling point TS6 is positively correlated with pH.

**Table 3:** Pearson correlation coefficient between pH and soil carbon, nitrogen and C/N ratio.

	TS1	TS3	TS4	TS5	TS6	TS7
TOC	−0.639**	−0.076	0.073	−0.638**	−0.571**	−0.800
TON	−0.412	−0.157	0.102	−0.097	0.667**	−0.915*
C/N	−0.497*	−0.193	0.074	−0.794**	−0.360**	−0.835
TIC	−0.506*	0.053	−0.889**	−0.679*	0.149	−0.737
TIN	−0.460*	0.273	−0.685*	−0.730**	−0.806**	−0.367

**Notes:**

* Correlation is significant at the 0.05 level

** Correlation is significant at the 0.01 level.

### Influence of soil texture to TOC and TON

**Figure 5:**
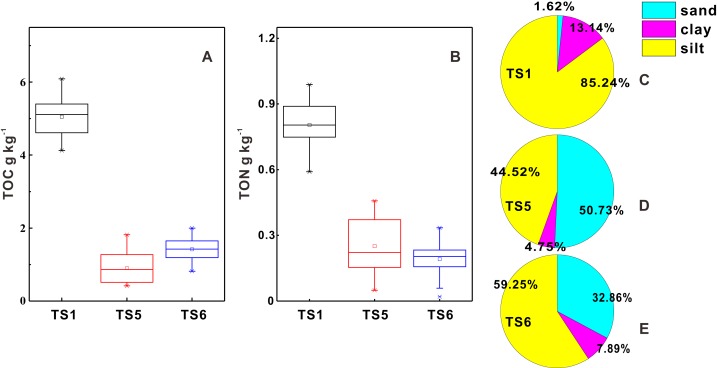
TOC and TON average contents and average percentages of clay, silt and sand of soil below 50 cm. (A) Box diagram of TOC contents; (B) box diagram of TON contents; (C–E) pie charts of clay, silt and sand.

It has been reported that the effects of land-use and vegetation are eliminated when soil depth reaches 50 cm (Slessarev et al., 2016). The TOC and TON average contents, as well as average percentages of clay, silt and sand, of soil below 50 cm were calculated to explore the influence of soil texture to organic matter (Fig. 5). Sampling points TS1, TS5 and TS6 were chosen because they are typical and different from each other in composition of clay, silt and sand. The results show that TOC average content at sampling point TS1 is about four times more than that at TS5 and TS6 while TON average content at TS1 is about two times more than that at TS5 and TS6. As shown in the pie chart, samples at TS1 are much richer in silt and clay than samples at TS5 and TS6.

## Discussion

### Reasons for pH distribution characteristics

Soil pH is an important soil chemical property which controls many soil properties and it is affected by climate, soil buffering system, plants, etc. (Hong, Gan & Chen, 2019). In this research region, pH values in topsoil are more affected by precipitation and evaporation: Abundant rainfall causes the leaching of calcium and magnesium ions in central region which lowers soil pH (Zhao et al., 2018b). But in the western and eastern region where rainfall is relatively lower, serious soil salinization occurs in sandy soil as a result of evaporation exceeding precipitation, which lead to higher soil pH values in these regions (Zhao et al., 2018a). In general, pH values in the topsoil are lower because topsoil is rich in organic matter and the decomposition of organic matter will lead to the production of more organic acids, thus lowering pH of topsoil (Hong, Gan & Chen, 2019). In some soil profiles, pH values tend to increase and then decrease along with soil depth, which is particularly obvious in TS4 and TS6. As shown in Table 1, the middle section of the soil profile at TS6 is rich in iron and manganese nodules, which is a combination of iron and manganese oxides essentially. These oxides react with hydrogen ions (H^+^) and thus the pH values would not be too low in that layer. In addition, there are some iron manganese nodules existing in the other soil profiles. The positive peaks of pH values in the middle section of soil profiles may be explained by the existence of these iron manganese nodules.

### Correlation between pH and C/N ratio

The C/N ratio can estimate the quality of soil organic matter, especially in the process of decomposition and nitrification (Dignac et al., 2002). Recently, some studies revealed that soil C/N ratio can significantly affect rice yield and indicated that microorganisms are responsible for almost all decomposition of organic matter in terrestrial ecosystems (Dai et al., 2019; Nicolardot, Recous & Mary, 2001; Gregorich et al., 1994). Soil pH is vital in determining the variation of microorganisms’ community structure and diversity (Tripathi et al., 2018), which will control the process of decomposition and nitrification. Therefore, there should be correlation between soil pH and C/N ratio theoretically. The data do show negative a relationship between them at sampling points TS1, TS5, TS6 and TS7. Moreover, soil pH values tend to increase along with soil depth; by contrast, C/N ratios tend to decrease along with soil depth generally. However, the correlations between them are very weak at sampling points TS2, TS3 and TS4. Future research is needed to explore the reason.

### Factors that influence soil carbon and nitrogen

Soil TOC and TON are important soil properties influenced by various kinds of factors. Tesfaye et al. (2016) reported that the concentrations of carbon and nitrogen are highly influenced by land use types and soil depth. Topsoil from croplands and forest lands has higher carbon and nitrogen concentrations than that from other kinds of lands in this study region, because it is plant residues and animal material that provide raw materials to produce TOC and TON in topsoil (Polyakov & Lal, 2004). The rice field (where sampling point TS4 is located) has been abandoned for 2 years. Carbon and nitrogen concentrations in topsoil at sampling point TS4 are much lower than those at TS1 and TS2, which could be a result from the lack of plant and animal residues input. On the other hand, with reduced organic matter input and increased decomposition rates, TOC and TON contents decrease along with soil depth (Han, Li & Tang, 2015).

Most soil samples are sandy loam and silt loam, which are rich in silt and sand. The TOC and TON average contents of samples from sampling point TS1 where soil is rich in silt and clay are much higher than those from TS5 and TS6 where soil is rich in sand. Therefore, on the condition that other factors are excluded, the silt and clay contents are positively correlated with TOC and TON contents. Hassink (1994) has reported that TOC contents were positively correlated with clay and silt contents in soil, because clay and fine silt particles help to stabilize soil organic matter (Baldock & Skjemstad, 2000). Theoretically, smaller particles have bigger specific surface areas, thus owning stronger ability to absorb organic matter and protect them from being decomposed by microorganisms.

Soil inorganic carbon is mainly stored in the residue of parent rocks or minerals that derive from the preservation or formation process of carbonates (Zissimos et al., 2019). Soil inorganic nitrogen can be mainly divided into NO_y_(NO_3_^−^) and NH_x_(NH_4_^+^), which can exert different impacts on soil chemical reactions (Tian & Niu, 2015). At some sampling points in the study region, the concentrations of TIC and TIN are negatively correlated with pH values generally. Soil TIC has been regarded as an important buffering system which strongly regulates soil pH values (Slessarev et al., 2016). Carbonates are the main provider of soil inorganic carbon and have relatively high pH values because they react with H^+^. Meanwhile, soil pH is controlled by different buffering systems whose effects on pH may be overlapped (Hong, Gan & Chen, 2019), which explain why the correlation between pH and TIC is very weak at some sampling points. Soil nitrogen cycles control a series of processes that produce and consume H^+^, causing the changes of soil acidity (Binkley & Richter, 1987). In terms of TIN, NH_4_^+^ ion and NO_3_^−^ ion pose different impacts on soil acidity (Tian & Niu, 2015). Specifically, NO_3_^−^ ion lowers soil pH by causing the leaching of Ca and Mg and reducing their concentrations (Lu et al., 2018; Tian & Niu, 2015), while NH_4_^+^ acidizes soil by exchanging base cations directly (Matschonat & Matzner, 1996). These properties of TIN explain the negative correlation between TIN and pH at some sampling points.

### Some suggestion to local farming method

Weeds and trees on the wasteland where sampling point TS5 is located were burned to prepare for cultivation. This is a slash-and-burn farming method which is common in local areas. Fire is a traditional tool to open “new land” for agriculture when the existing land lost its fertility and “the old” land will be abandoned for growing plants and replenishing soil fertility (Ketterings et al., 1999). However, it will bring many adverse impacts to environment, peasant and urban societies (Arroyo-Kalin, 2012; Nardoto & Da Cunha Bustamante, 2003). More efficient measures are needed to increase soil fertility and mull over the problems. The sugarcane field where sampling point TS2 is located has large areas of sugarcane. Growing in central, northern and north-eastern Thailand, sugarcane is one of the main crops that have great impacts on the Thai economy (Prasara & Gheewala, 2016). Kumar et al. (2010) revealed that after the sugarcane production process, there are still a considerable amount of waste which can be converted into organic manure. Microorganisms and subsequent vermicomposting can accelerate the converting process (Kumar et al., 2010). Therefore, local inhabitants should change the primitive farming method to a more efficient and environmentally friendly one.

## Conclusions

In the Mun River Basin, TOC and TON contents in topsoil of agricultural lands and forest lands are higher than those of other kinds of lands. Vertically, distributions of TOC and TON contents in the soil profiles are influenced by soil texture significantly. Specifically, the soil containing more silt and clay has higher TOC and TON contents because clay and silt particles have bigger specific surface areas to absorb and protect organic matter. Soil C, N and C/N ratio tend to be negatively correlated with soil pH in most soil profiles, indicating the close relationship between soil pH and soil C and N.

## Supplemental Information

10.7717/peerj.7880/supp-1 Supplemental Information 1 Raw data of soil C,N, C/N ratio, pH and percentages of clay, silt and sand.

## Additional Information and Declarations

### Data Availability

The following information was supplied regarding data availability: The raw data is available in the Supplemental File.
